# Advantages and Limitations of SNP Array in the Molecular Characterization of Pediatric T-Cell Acute Lymphoblastic Leukemia

**DOI:** 10.3389/fonc.2020.01184

**Published:** 2020-07-17

**Authors:** Monika Lejman, Monika Włodarczyk, Borys Styka, Agata Pastorczak, Joanna Zawitkowska, Joanna Taha, Łukasz Sędek, Katarzyna Skonieczka, Marcin Braun, Olga Haus, Tomasz Szczepański, Wojciech Młynarski, Jerzy R. Kowalczyk

**Affiliations:** ^1^Laboratory of Genetic Diagnostics, Department of Pediatric Hematology, Oncology and Transplantology, Medical University of Lublin, Lublin, Poland; ^2^Laboratory of Genetic Diagnostics, University Children's Hospital, Lublin, Poland; ^3^Department of Pediatric, Oncology, Hematology and Diabetology, Medical University of Łódz, Łódź, Poland; ^4^Department of Pediatric Hematology, Oncology and Transplantology, Medical University of Lublin, Lublin, Poland; ^5^Department of Microbiology and Oncology, Medical University of Silesia in Katowice, Katowice, Poland; ^6^Department of Clinical Genetics, Faculty of Medicine, Collegium Medicum in Bydgoszcz, Nicolaus Copernicus University in Torun, Bydgoszcz, Poland; ^7^Department of Pathology, Chair of Oncology, Medical University of Łódz, Łódź, Poland

**Keywords:** childhood, T-cell acute lymphoblastic leukemia, SNP array, CNAs, molecular characterization

## Abstract

T-cell acute lymphoblastic leukemia (T-ALL) is a highly heterogeneous disease, and numerous genetic aberrations in the leukemic genome are responsible for the biological and clinical differences among particular ALL subtypes. However, there is limited knowledge regarding the association of whole-genome copy number abnormalities (CNAs) in childhood T-ALL with the course of leukemia and its outcome. The aim of this study was to identify the pattern of whole-genome CNAs in 86 newly diagnosed childhood T-ALL cases using a high-density single-nucleotide polymorphism array. We analyzed the presence of whole-genome CNAs with respect to immunophenotype, clinical features, and treatment outcomes. A total of 769 CNAs, including trisomies, duplications, deletions, and segmental loss of heterozygosity, were detected in 86 analyzed samples. Gain or loss of chromosomal regions exceeding 10 Mb occurred in 46 cases (53%), including six cases (7%) with complex chromosomal alterations. We observed that microdeletions in selected genes (e.g., *FIP1L1* and *PDGFRB*) were related to the clinical features. Interestingly, 13% of samples have a duplication of the two loci (*MYB* and *AIH1—*6q23.3), which never occurred alone. Single-nucleotide polymorphism array significantly improved the molecular characterization of pediatric T-ALL. Further studies with larger cohorts of patients may contribute to the selection of prognostic CNAs in this group of patients.

## Introduction

T-cell acute lymphoblastic leukemia (T-ALL) accounts for ~15% of pediatric ALL cases (1). T-cell acute lymphoblastic leukemia is a biologically heterogeneous malignancy with numerous genetic aberrations in the leukemic genome. T-cell acute lymphoblastic leukemia has been divided into four subtypes according to the European Group for the Immunological Classification of Leukemia (EGIL): pro-T EGIL T-I (cCD3^+^, CD7^+^), pre-T EGIL T-II (cCD3^+^, CD7^+^, and CD5/CD2^+^), cortical T EGIL T-III (cCD3^+^, Cd1a^+^, sCD3^+/−^), and mature-T EGIL T-IV (cCD3^+^, sCD3^+^, CD1a^−^) (2). Early T-cell precursor (ETP)–ALL is characterized as an additional subtype of T-cell ALL with blasts usually negative for CD1a and CD8, weak expression of CD5, and the presence of one or more myeloid or stem cell markers (3, 4). The prognostic factors and risk stratification are different in T-ALL when compared to B-lineage leukemia. Minimal residual disease (MRD) response plays an essential role in the risk group assignment; leukocytosis and age at diagnosis are not independent prognostic factors in T-ALL (5). Generally, pre-T ALL shows a worse outcome than pre-B ALL in both pediatric and adult patients (6). Currently, risk-adapted therapy and appropriate supportive care result in a relatively high 5 year overall survival rate of 80%. However, effective treatment of T-ALL relapses is still clinically challenging (5). Because most patients with relapse were originally stratified into an intermediate risk group, none of the existing prognostic genetic markers were efficient enough to predict treatment outcome in T-ALL. In the last 10 years, advanced genomic and transcriptomic studies using high-throughput techniques have provided a better understanding of the pathogenesis of T-ALL (7, 8). Moreover, associations of molecular lesions with specific T-ALL subtypes, clinical features, and outcomes have been documented (9).

However, there is still limited knowledge about the association of whole-genome copy number abnormalities (CNAs) in childhood T-ALL with disease course and outcome. In this study, we analyzed the pattern of whole-genome CNAs using a high-density single-nucleotide polymorphism (SNP) array in childhood ALL, depending on the maturation state of leukemic cells, clinical features, and treatment outcome.

## Materials and Methods

### Patients

A series of bone marrow aspirates from 86 children with newly diagnosed T-ALL were analyzed prior to any oncological treatment. All patients were treated in 13 centers of the Polish Pediatric Leukemia/Lymphoma Study Group, according to the following protocols: ALL IC BFM 2002 [*n* = 15 (17%)], ALL IC BFM 2009 [*n* = 56 (66%)], and AIEOP BFM ALL 2017 [*n* = 15 (17%)], within the randomized trial of the International Berlin-Frankfurt-Munster Study Group (I-BFM-SG) for the therapy of childhood ALL. Bone marrow samples from all patients were cytogenetically investigated by G-banding analysis at the time of diagnosis. Targeted fluorescence *in situ* hybridization (FISH) *t*(9,22) (q34; q11)/*BCR-ABL1*, 11q23/*KMT2A* rearrangements were performed in 86 cases (100%). Genetic tests were performed for all ALL samples. The majority of patient samples (49 of 86) were examined in the Department of Diagnostic Genetics of Medical University of Lublin, and these samples were collected from the Department of Hematology, Oncology, and Transplantology in Lublin. The remaining data on patient cytogenetics were previously established within the therapeutic program through a combined cytogenetics FISH screening in the National Reference Center: Department of Clinical Genetics, Collegium Medicum in Bydgoszcz. Immunophenotypic data enabled the classification of patients into pre-T ALL [*n* = 24 (28%)], cortical T-ALL [*n* = 23 (26.6%)], mature T-ALL [*n* = 21 (24%)], and ETP-ALL [*n* = 2 (2%)] based on the EGIL criteria. In 16 cases (19%), immunophenotypic data were not available to determine a specific subtype of T-ALL. The clinical and laboratory details of patients with T-ALL are presented in Table 1. All of the participants have written consent to participate and publish the data. All procedures performed in studies involving human participants were in accordance with the ethical standards of the institutional and/or national research committee and with the 1964 Helsinki Declaration and its later amendments or comparable ethical standards. Informed consent was obtained from all individuals included in the study and their parents or guardians on behalf of any participant younger than 16 years. The study was approved by the ethics committee (KNW/0022/KB1/153/I/16/17).

**Table 1:** Clinical and biological characteristics of the study group with respect to the implemented treatment protocol.

		**ALL IC-BFM 2002**		**ALL IC-BFM 2009**		**AIEOP BFM ALL 2017**		***p*-value**
		***n*** **= 15**		***n*** **= 56**		***n*** **= 15**		
Age at diagnosis (years)		9.34 (2.84–17.53)		7.68 (1.08–16.34)		10.06 (2.41–17.67)		0.047
Platelets		100,857.1 (12–000)		110,743.9 (273–490,000)		603,42.21 (60–520,000)		0.057
Hg [g/dl]		9.72 (4.30–15.8)		9.94 (4.7–15.0)		10.84 (7.7–13.0)		0.151
Blasts—peripheral blood (%)		16.16 (0.00–96.0)		17.49 (0.0–96)		20.72 (3.0–80.0)		0.595
Blasts—bone marrow (%)		79.57 (36.0–100)		82.35 (80.0–100)		79.84 (30.0–100)		0.336
WBC count (n/μL)		127,863 (220–791,490)		142,861 (8,687–791,490)		100,144 (220–403,160)		0.490
Sex	Female	4	27%	17	30%	6	40%	0.705
	Male	11	73%	39	70%	9	60%	
Poor steroid response	No	5	33%	32	57%	12	80%	0.155
	Yes	10	67%	24	43%	3	20%	
MRD FMC 15 (%)	<10	10	67%	32	58%	9	60%	0.838
	>10	5	33%	23	42%	6	40%	
Hyperdiploidy	No	13	87%	55	98%	14	93%	0.156
	Yes	2	13%	1	2%	1	7%	
*KMT2A+*	No	13	87%	52	93%	14	93%	0.710
	Yes	2	13%	4	7%	1	7%	
Karyotype	Normal	10	67%	31	62%	12	80%	0.124
	Complex	4	27%	19	38%	3	20%	
	Hypertriploidy	1	7%	0	0%	0	0%	
Mediastinal tumor	No	8	57%	33	59%	6	43%	0.553
	Yes	6	43%	23	41%	8	57%	
Hepatomegaly	No	4	27%	22	39%	5	33%	0.755
	Yes	11	73%	34	61%	10	67%	
Splenomegaly	No	3	20%	22	39%	4	27%	0.398
	Yes	12	80%	34	61%	11	73%	
Lymph node involvement	No	11	79%	44	79%	13	93%	0.462
	Yes	3	21%	12	21%	1	7%	
Risk group	Non-HR	9	64%	26	47%	8	53%	0.761
	HR	5	36%	29	53%	7	47%	
HSCT	No	10	71%	35	70%	14	93%	0.181
	Yes	4	414%	15	30%	1	7%	
Relapse	No	12	86%	44	86%	14	93%	0.749
	Yes	2	14%	7	14%	1	7%	
Follow up (years)		9.32		3.87		0.63		**0.001**

*WBC, white blood count; MRD FMC, minimal residual disease flow cytometry; HSCT, hematopoietic stem cell transplantation; HR, high-risk group; non-HR, non–high-risk group*.

*Data are presented as medians and IQRs or numbers and percentages. The follow-up time did not differ significantly between the two protocols, ALL IC BFM 2002 (9.32 years) and ALLIC BFM 2009 (8.37 years), but was shorter for AIEOP BFM ALL 2017 (p <0.01). Apart from age at diagnosis, no other significant differences with respect to the clinical and biological features between protocols were observed. Differences between protocols were assessed using the Kruskal–Wallis or the χ^2^ tests. The bold value is considered statistically significant*.

### Laboratory Methods

Bone marrow samples were aspirated into anticoagulant (EDTA)–containing tubes from patients with T-ALL at the time of diagnosis. Genomic DNA was isolated with the QIAamp DNA Blood Mini Kit (Qiagen, Hilden, Germany). DNA samples were stored at −20°C until SNP arrays experiments were performed. The median leukemic cell counts in the collected bone marrow were assessed using flow cytometry to be 82.43% (range = 30–100%). The concentration and quality of isolates were determined by spectrophotometry (NanoDrop 8000; Thermo Fisher Scientific, Waltham, MA, USA). The microarray analyses were performed with the use of a CytoScan HD array [2,670,000 markers, including 750,000 SNP and 1,900,000 non-polymorphic copy number variant (CNV) markers] (Applied Biosystems, part of Thermo Fisher Scientific). All laboratory procedures were carried out according to the manufacturer's protocols. A previously described microarray method that has been standardized in our laboratory based on AIEOP-BFM array screening strategy recommendations was used for microdeletion and microduplication assessments (10). In total, 250 ng of genomic DNA was analyzed in accordance with the manufacturer's protocols. The study was based on an analysis of scanned data files that were generated with the Chromosome Analysis Suite v 3.3 (ChAS; Thermo Fisher Scientific). Furthermore, the copy numbers of altered regions (CNAs) were calculated, and the data were normalized to a reference model (Thermo Fisher Scientific) of baseline reference intensities, NA 33 (hg19/CRCh37). The copy number states and their breakpoints were determined with the use of the hidden Markov model software package. The threshold levels of log_2_ ratio ≥ 0.5 and ≤0.5 were used for the categorization of the altered chromosomal regions as CNV gains and losses, respectively. The identification of normal diploid markers in the cancer samples constituted an essential part of the algorithm, which was particularly significant in highly sample-induced aberrations. Furthermore, unaltered diploid markers were used for the calibration of signals, resulting in a log_2_ ratio of 0 (e.g., copy number 2). The algorithm also indicated that the identified unaltered diploid markers corresponded with CN = 4. In such a case, the log_2_ ratio was readjusted, and the chromosomal ploidy of 4 was reported. The obtained data were analyzed based on two different criteria: genome-wide CNVs and leukemia-associated region/gene-specific CNAs (leukemia genes_all_20150505; Fullerton Overlap Map_hg19). Our panel of 92 selected genes was created based on comprehensive literature describing genetic alterations and their role in pathogenesis T-ALL (7, 9, 11, 12). The genes are listed in Table S1. The minimal number of probes was applied to determine the CNAs were 50 probes for duplication (gain) and 25 probes for deletion (loss). Copy number–neutral loss of heterozygosity (LOH) is reported when the length is >3,000 kbp. To further identify the genes involved in the CNVs, two databases were applied: the UCSC database (http://genome.ucsc.edu) and Ensemble (http://www.ensembl.org). For microarray results, copy number polymorphisms were excluded based on comparison with the Database of Genomic Variants (http://projects.tcag.ca/variation/).

### Statistical Analysis

Clinical parameters, such as patient's age at diagnosis, white blood count (WBC), hemoglobin, and platelet count at diagnosis, percentage of blasts in peripheral blood and bone marrow, MRD level, and survival parameters, are presented as medians with interquartile ranges (IQRs) and were compared using Kruskal–Wallis analysis of variance or the χ^2^ test. The association between CNAs and clinical features was tested using the χ^2^ test or Fisher exact test (when the expected values were <5). Individual *p*-values corrected for multiple comparisons of all 92 genes (Bonferroni correction) are reported. Additionally, for genes in which correlations of CNAs with clinical features were significant, a multivariate logistic regression model was used with CNVs as the outcome variable and clinical features as predictors. *p* < 0.05 were considered statistically significant. Statistical analysis was performed using Statistica Software version 13.1 PL (StatSoft, Krakow, Poland) and R software, version 3.5.4.

## Results

### Characterization of the Study Group

The group consisted of 27 girls (32%) and 59 boys (68%). The age of patients ranged from 1.08 to 17.67 years (median age, 8.37 years). The characteristics of the patient cohort are presented in Table 1.

### Molecular Karyotypes of Childhood T-ALL

**Figure 1:**
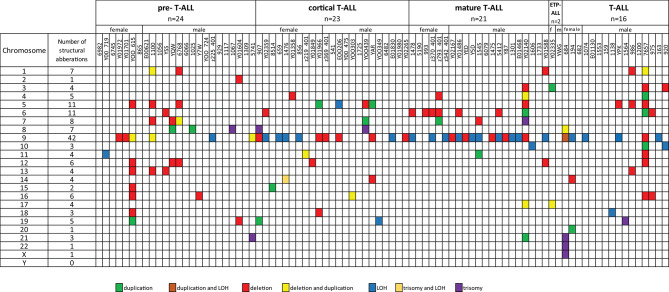
Frequencies of structural chromosomal abnormalities in 86 T-ALL cases according to particular T-ALL subtypes.

Fifty-five T-ALL cases showed normal karyotype, and in six cases, there was a failure of bone marrow cell culture in cytogenetic analysis. Original karyotypes and revisions based on SNP array analysis in 86 pediatric patients with T-ALL are presented in Table S2. Duplication or deletion of chromosomal regions exceeding 10 Mb, which are potentially cytogenetically visible, occurred in 46 cases (53%), including six cases (7%) with complex chromosomal alterations. In 16 cases (18%), alterations were limited only to homozygosity regions (hmz). No monosomies or cases of whole-chromosome LOH were found. Trisomies of whole chromosomes 7, 8, 13, 14, 19, 21, 22, and X were identified in eight cases (9%): one trisomy of chromosomes 7, 14, 19, 22, and X; two trisomies of chromosome 21; and three trisomies of chromosome 8. One patient presented trisomies in chromosomes 21, 22, and X. No structural abnormalities were found in 27 patients (31%) (Figure 1).

Additionally, in seven cases (8%), we noticed inconsistency between the cytogenetic and SNP array testing results. In general, array testing added novel or complementary genetic information to the cytogenetic results by better defining chromosome break points in 66 cases (77%). The smallest partial gains of overlapping regions were observed on chromosomes 9q 118,514,469–141,020,389 bp (9q33.1–q34.3), 5p 113,576–44,151,712 bp (5p15.33–p12), 7q 148,013,257–159,119,707 bp (7q36.1–q36.3), and 8q 131,101,950–146,295,771 bp (8q21–q24.3). The smallest regions of overlap of recurrent deletions were 9p 203,861–35,701,152 bp (9p11–p21.2) and 9p 203,861–35, 701,152 bp (9p21.1–pter), 6q 79,110,264–110,247,755 bp (6q14.1–q21), 5q 100,821,228–180,719,789 bp, (5q21.3–qter), 1p 849,466–8,096,240 bp 1(p36.33–p36.31), 7q 108,354,488–159,119,707 bp (7q31.1–qter), 12p 173,786–31,598,670 bp (12p13.31–p11.21), 13q 40,010,662–70,747,804 bp (13q13q21.3), and 16q 57,712,418–88,818,573 bp (16q21–q24.3). Twenty-eight cases of segmental LOH were found in 27 cases. The most common region for LOH was 9p 192,128–21,894,495 bp (9p24.3p13.3), identified in 21 cases. In the described cohort, the presence of alterations in chromosome 9 was significantly associated with a specific immunophenotype: mature T-ALL (68.75% of cases), whereas pre–T-ALL was characterized by a lack of abnormalities in this chromosome (83.33% of cases), *p* = 0.019.

**Figure 2:**
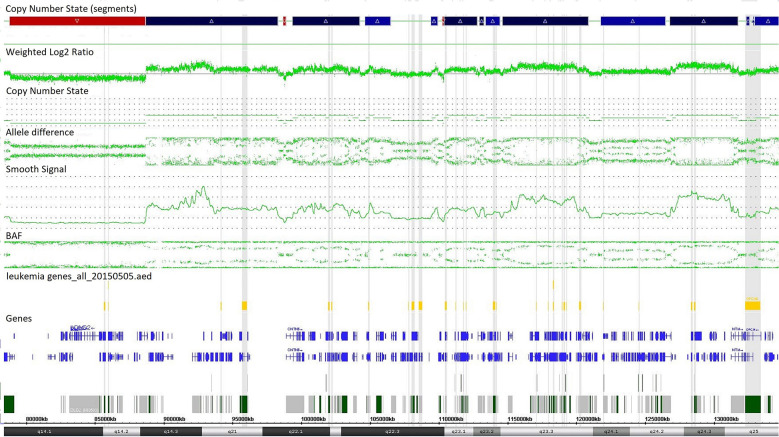
Complex copy number gains and losses in region 11q14.1q25 of chromosome 11 in patient with T-cell ALL by single-nucleotide polymorphism (SNP) array (case z219-401). Colored segmental regions (top line: red color—deletions; blue color—gains), copy number state allele difference, and smooth signal (bottom panel) aberrations by SNP array.

Single-nucleotide polymorphism array testing revealed multiple alternating changes (normal segments, gains, and losses) within the 11q14.1q25 region of chromosome 11 in patient z219–401 (Figure 2). These alterations were also observed in cytogenetic analysis as triplications between 11q13 and 11q12 of chromosome 11.

**Figure 3:**
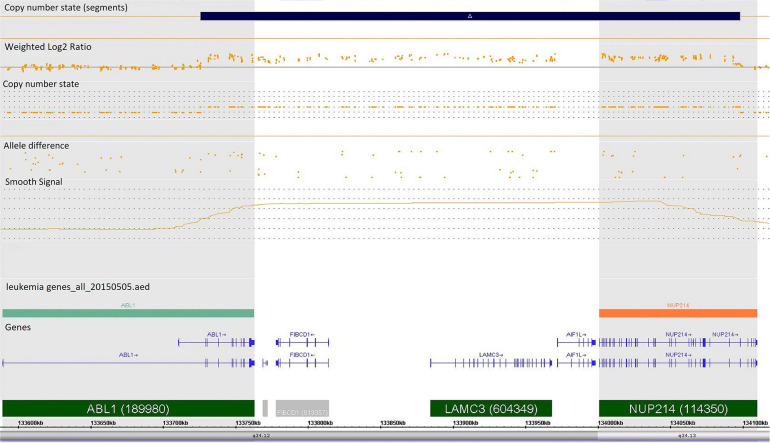
Single-nucleotide polymorphism (SNP) array of the amplification in 9q34 was limited by NUP214 (from exon 1 to exon 31) and ABL1 (from exon 2 to the 5′ end of the gene). Colored segmental region (top line: blue color—gains), copy number state allele difference, and smooth signal (bottom panel) aberrations by SNP array.

**Figure 4:**
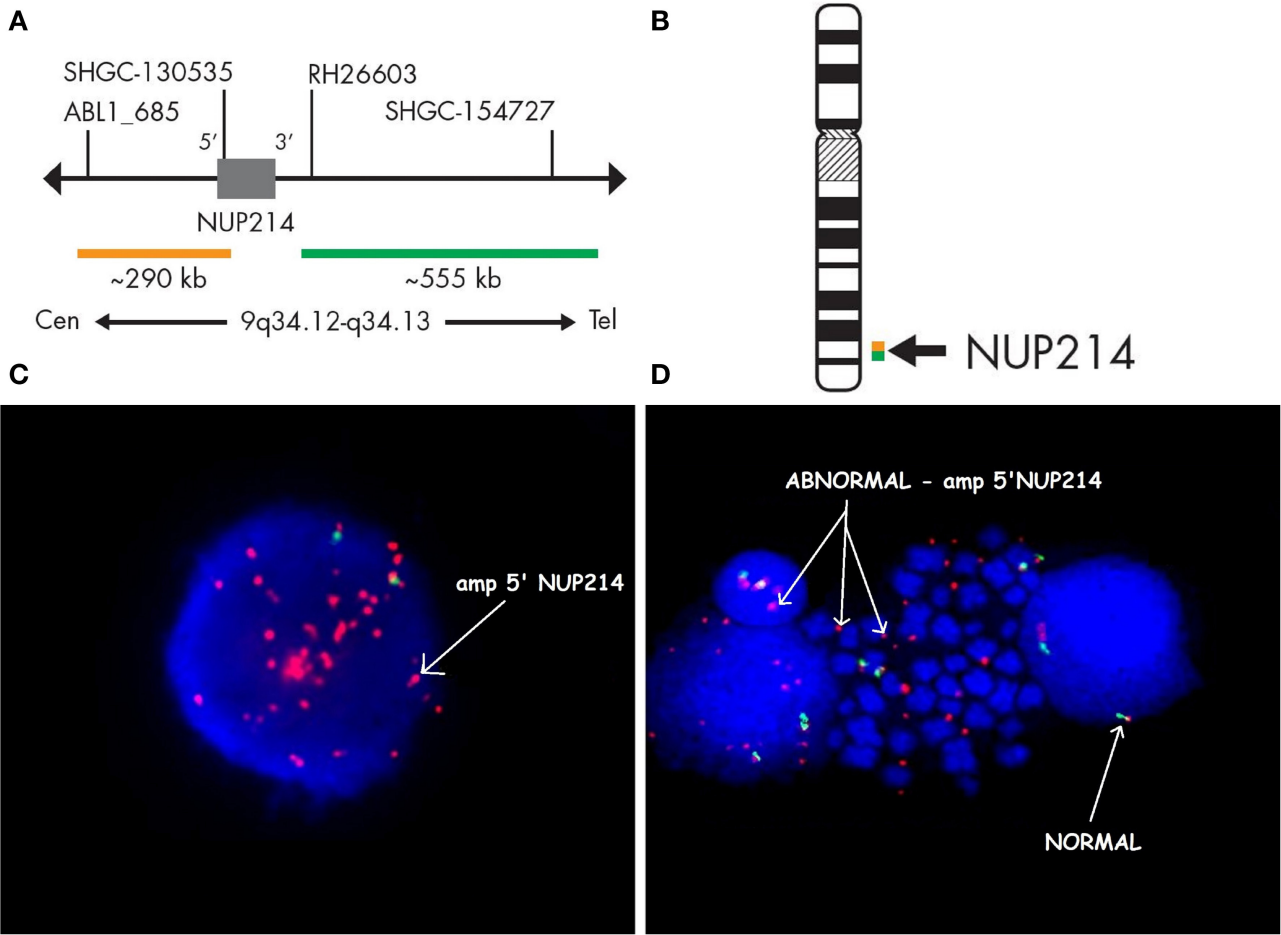
Fluorescence *in situ* hybridization revealing an amplification of NUP214. SPEC NUP214 probe map **(A)**. Ideogram of chromosome 9 indicating the hybridization locus **(B)**. Images of the FISH results with the ZytoLight-SPEC NUP214 Dual Color Break Apart Probe revealing NUP214 amplification **(C,D)**. FISH was performed on both interphase **(C)** and metaphase cells **(D)**. Cells displaying *NUP214-ABL1* amplification are shown (arrows). Normal cells exhibited two red/green signals (arrows). Episomes are represented as small dots between chromosomes **(D)**.

Case 986 presented an amplification at 9q34 with breakpoints in the NUP214 and ABL1 genes (Figure 3). All abnormalities appeared to be present in the entire leukemic population. Interphase FISH testing confirmed the amplification of NUP214 (Figure 4).

### The Landscape of CNAs in Childhood T-ALL

**Figure 5:**
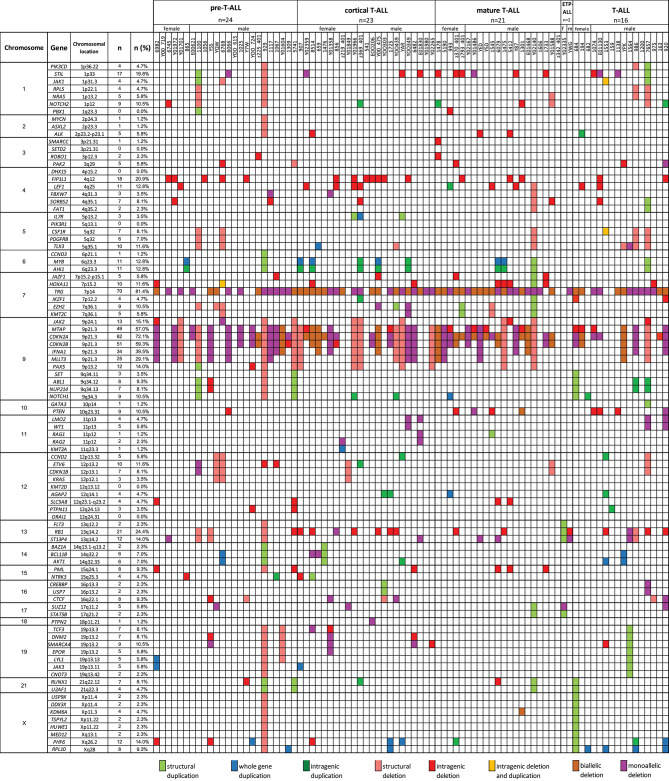
The frequencies of CNAs of all 92 selected genes in 86 T-ALL cases according to particular chromosomes.

A total of 769 CNAs, including trisomies, duplications, deletions, and segmental loss of heterozygosity, were detected in 86 analyzed samples (Figure 5). The median number of CNAs per case was 16 (mean = 16.4, range = 1–49). Copy number abnormalities >10 Mb and <10 Mb accounted for 41% (10 trisomies, 75 duplications, 200 deletions, and 28 LOHs) and 59% (28 duplications of the whole gene, 42 intragenic duplications, 185 whole-gene monoallelic deletions, 85 whole-gene biallelic deletions, and 109 intragenic monoallelic deletions of gene and seven intragenic biallelic deletions), respectively.

**Figure 6:**
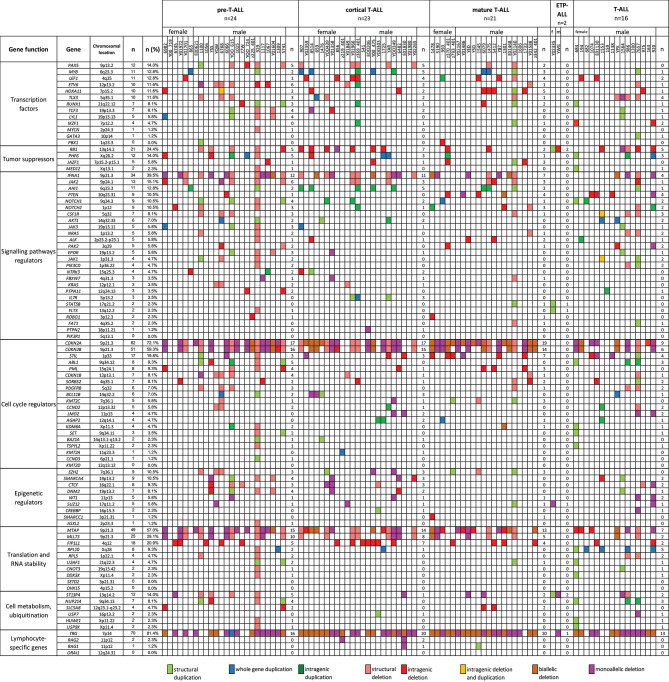
The frequencies of CNAs of all 92 selected genes in 86 T-ALL cases according to particular T-ALL subtypes. Owing to the small amount of data on the CNA frequency observed in T-ALL (data reported thus far are focused on the mutation status of analyzed genes), it is unclear what effects individual changes have on the pathogenesis and course of T-ALL.

We also selected a panel of 92 genes with documented roles in the pathogenesis of T-ALL and searched for CNAs within this gene panel. The results of the analysis are presented in Figure 6. In our study, CNAs in *MYB* and *AIH1* (6q23.3) co-occurred in 11 cases (12.94%). We noticed whole-gene duplications associated with chromosomal alterations in 3 of 11 cases and intragenic duplications (from exon 3 to the 3′ end) in 2 of 11 cases. In the remaining cases, we noticed coexisting whole-gene duplications in *MYB* and intragenic duplications in *AIH1* (from exon 27 to the 3′ end). More importantly, CNAs in *MYB* and *AIH1* did not occur separately in any of our samples (Figure 5). In the case of *DNM2* (19p13.2), CNA occurred in seven cases (8.24%). In two cases, we found duplications associated with chromosomal alterations, and in five cases, we found deletions, including two cases with deletion associated with chromosomal alteration, one monoallelic deletion, and two with intragenic monoallelic deletions (from exon 2 to 3′ end). Two cases of intragenic deletions in *ROBO1* (3p12.3) were found in this study. Three cases presented deletion of exon 2 in *PAK2* (3q29). In 18 samples (21.18%), intragenic deletions (from exon 6 to exon 10) in *FIP1L1* (4q12) were identified. The monoallelic intragenic deletion (from upstream region 1 exon to 3 exon) occurred in *LEF1* (4q25) in three cases. Monoallelic intragenic deletions covering the first exon were observed in *HOXA11*-7p15.2 (nine cases), in *JAZF1-*7p15.2-p15.1 (two cases), *PTEN*-10q23.31 (two cases), and in *SLC5A8*-12q23.1 (four cases). Monoallelic intragenic deletions of exons 3 and 4 were observed in six cases of *PML* (15q24.1). Moreover, monoallelic intragenic deletions involving the region from exon 16 to exon 22 of gene *SORBS2* were found in five cases. Monoallelic intragenic deletions covering the last exon of genes were found in *PTEN*-10q23.31 (from exon 2 to 3′ end in three cases) and *STIL-1* (from exon 8 or 12 to 3′ end in nine cases).

### Association of CNAs With Clinical Features and Response to Treatment

**Figure 7:**
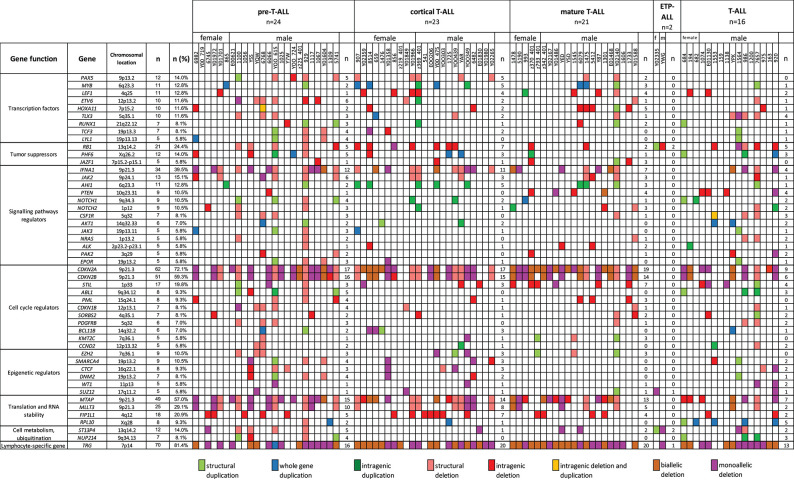
Overview of molecular alterations identified in 86 samples according to particular subtypes of T-ALL. Heat map represents genes in which CNAs have been identified in at least 5% of patients.

**Figure 8:**
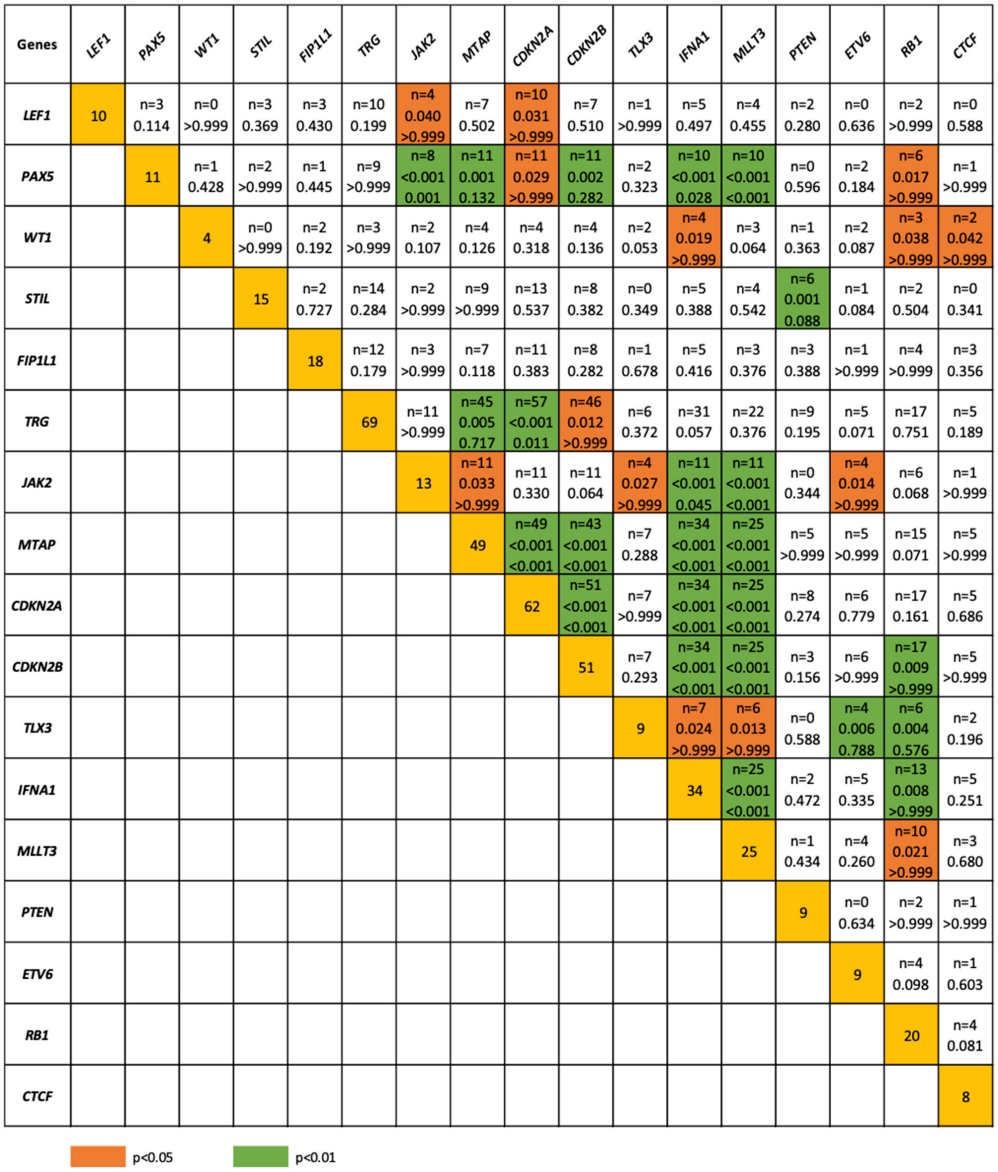
Concomitance of gene deletions in 86 children with T-ALL. Yellow boxes indicate the total number of patients with a deletion. Other boxes indicate the number of patients with two specific abnormalities. Orange and blue colors indicate significant overlap associations in individual comparisons (*p* < 0.01 or *p* < 0.05), respectively. If association in individual comparison of two genes was significant, *p*-value corrected for multiple comparisons was added.

**Figure 9:**
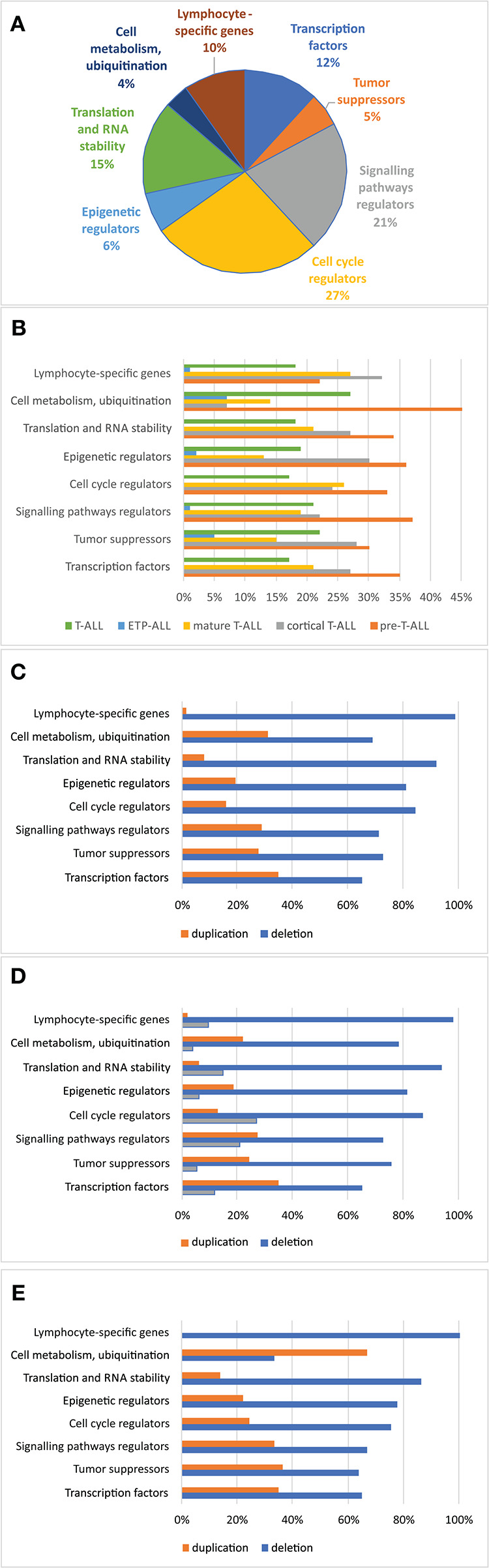
Frequency of copy number alterations identified in 86 T-ALL cases. The incidence of CNAs relative to specific groups of genes **(A)**. Percentages of CNAs relative to specific groups of genes **(B)**. Percentages of CNAs relative to specific group of genes in the group of boys **(C)**, girls **(D)**, and all cases **(E)**.

The relationship between microdeletions and microduplications of targeted genes (in which CNAs have been identified in at least 5% of patients), their functions, and immunophenotype is shown in Figure 7. Moreover, in some cases, multiple gene deletions occurred in the same patients. The following interactions were still significant after correction for multiple testing: *CDKN2A-CDKN2B, CDKN2B-IFNA1, CDKN2B-MLLT3, PAX5-MLLT3, MTAP-CDKN2A, MTAP-CDKN2B, CDKN2A-IFNA1, IFNA1-MLLT3, MTAP-IFNA1, MTAP-MLLT3, PAX5-JAK2, JAK2-MLLT3, TRG-CDKN2A, PAX5-IFNA1, CDKN2A-MLLT3, JAK2-IFNA1, PAX5-MTAP, PAX5-CDKN2B, TRG-MTAP, STIL-PTEN, TRG-CDKN2B, JAK2-ETV6, PAX5-RB1* (Figure 8). The frequency of CNAs was analyzed depending on the pathways and gene functions: transcriptional and epigenetic activity, regulation of the cell cycle, and translation or RNA stability (Figure 9). We found no significant predominance of alterations in any of the tested genes. The biggest difference was apparent for deletion of *CSF1R*, which was more prevalent in boys than in girls (13.6% vs. 0.0%), but this difference was not statistically significant (*p* = 0.05237 without correction for multiple comparisons). We observed a significant difference in *NOTCH2* (*p* = 0.010), *ALK* (*p* = 0.016), *LEF1* (*p* = 0.023), *TRG* (*p* = 0.001), and *SLC5A8* (*p* = 0.023) deletion frequencies in groups of patients with different WBC levels (*p* < 0.05). In our cohort, we found a difference in *FIP1L1* (*p* = 0.004), *LEF1* (*p* = 0.048), *CSF1R* (*p* = 0.048), *PDGFRB* (*p* = 0.025), and *TLX3* (*p* = 0.035) deletion frequency in a group of patients with poor response to steroids, compared to a group with good response (*p* < 0.05). We noticed a significant difference in *FIP1L1* (*p* = 0.015), *LEF1* (*p* = 0.007), *MTAP* (*p* = 0.005), and *CDKN2A* (*p* = 0.016) deletion frequencies in the high-risk group of T-ALL patients in comparison to non-high-risk group (*p* < 0.05). We also observed a difference in *FIP1L1* (*p* = 0.001), *LEF1* (*p* = 0.044), *PDGFRB* (*p* = 0.034), *CDKN2A* (*p* = 0.027), *CDKN2B* (*p* = 0.020), and *STAT5B* (*p* = 0.008) deletion frequency in group of patients with MRD flow cytometry (MRD FMC) levels on day 15 below 10%, compared to the group of patients with MRD FMC levels on day 15 exceeding 10% (*p* < 0.05). We found a significant difference in *FIP1L1* (*p* = 0.003), *WT1* (*p* = 0.014), and *PHF6* (*p* = 0.043) deletion frequency in the group of patients who relapsed in comparison to the group of patients who did not relapse (*p* < 0.05). We observed a difference in *MTAP* (*p* = 0.003), *IFNA1* (*p* = 0.007), and *MLLT3* (*p* = 0.008) deletion frequency in the group of younger patients (<10 years old), compared to the group of older patients (<10 years old) (*p* < 0.05) (Table 2). After adjusting for the multiple comparisons (92 genes), none of the associations between CNAs and clinical features analyzed was statistically significant.

**Table 2:** A comparison between the presence of deletions in particular genes and clinical features of T-ALL patients.

**(1)**	**WBC**			***p*-value**	**Adjusted**
**Gene**	**<5/L (*n* = 46)**	**5–10/L (*n* = 36)**	**>10/L (*n* = 4)**		***p*-value**
*NOTCH2*	9 (19.6)	0 (0.0)	0 (0.0)	0.010	0.939
*ALK*	2 (4.3)	1 (2.8)	2 (50.0)	0.016	>0.999
*LEF1*	2 (4.3)	8 (22.2)	1 (25.0)	0.023	>0.999
*TRG*	31 (67.4)	35 (97.2)	4 (100.0)	0.001	0.126
*SLC5A8*	0 (0.0)	3 (8.3)	1 (25.0)	0.023	>0.999
**(2)**	**Response to steroids**			***p*****-value**	**Adjusted**
**Gene**	**Poor (*****n*** **= 32)**	**Good (*****n*** **= 54)**			***p*****-value**
*FIP1L1*	12 (37.5)	6 (11.1)		0.004	0.335
*LEF1*	1 (3.1)	10 (18.5)		0.048	>0.999
*CSF1R*	6 (18.8)	2 (3.7)		0.048	>0.999
*PDGFRB*	5 (15.6)	1 (1.9)		0.025	>0.999
*TLX3*	7 (21.9)	3 (5.6)		0.035	>0.999
**(3)**	**Risk group**			***p*****-value**	**Adjusted**
**Gene**	**HR (*****n*** **= 43)**	**Non-HR (*****n*** **= 43)**			***p*****-value**
*FIP1L1*	14 (32.6)	4 (9.3)		0.015	>0.999
*LEF1*	1 (2.3)	10 (23.3)		0.007	0.663
*MTAP*	20 (46.5)	29 (67.4)		0.050	>0.999
*CDKN2A*	26 (60.5)	36 (83.7)		0.016	>0.999
**(4)**	**MRD FMC 15 day**			***p*****-value**	**Adjusted**
**Gene**	**<10% (*****n*** **= 52)**	**>10% (*****n*** **= 34)**			***p*****-value**
*FIP1L1*	5 (9.6)	13 (38.2)		0.001	0.131
*LEF1*	10 (19.2)	1 (2.9)		0.044	>0.999
*PDGFRB*	1 (1.9)	5 (14.7)		0.034	>0.999
*CDKN2A*	42 (80.8)	20 (58.8)		0.027	>0.999
*CDKN2B*	36 (69.2)	15 (44.1)		0.020	>0.999
*STAT5B*	0 (0.0)	2 (5.9)		0.008	0.735
**(5)**	**Relapse**			***p*****-value**	**Adjusted**
**Gene**	**No (*****n*** **= 75)**	**Yes (*****n*** **= 11)**			***p*****-value**
*FIP1L1*	12 (16.0)	6 (54.5)		0.003	0.307
*WT1*	2 (2.7)	3 (27.3)		0.014	>0.999
*PHF6*	8 (10.7)	4 (36.4)		0.043	>0.999
**(6)**	**Age**			***p*****-value**	**Adjusted**
**Gene**	**<10 years (*****n*** **= 61)**	**>10 years (*****n*** **= 25)**			***p*****-value**
*MTAP*	41 (67.2)	8 (32.0)		0.003	0.235
*IFNA1*	30 (49.2)	4 (16.0)		0.007	0.626
*MLLT3*	23 (37.7)	2 (8.0)		0.008	0.720

*Data are presented as n (%), χ^2^ test, or Fisher exact test. Only genes with p <0.05 are presented. Adjusted p-value for multiple comparisons: >0.999 for remaining genes*.

*WBC, white blood count; MRD FMC, minimal residual disease flow cytometry; HR, high-risk group; non-HR, non–high-risk group*.

*Data are presented as n (%), χ^2^ test, or Fisher exact test. Only genes with p <0.05 are presented: (1) p-value adjusted for multiple comparisons (92 genes): p > 0.999 for ALK, LEF1, SLC5A8, p = 0.939 for NOTCH2, p = 0.126 for TRG; (2) P-value adjusted for multiple comparisons (92 genes): p = 0.335 for FIP1L1, p > 0.999 for remaining genes; (3) p-value adjusted for multiple comparisons (92 genes): p = 0.663 for LEF1, p > 0.999 for remaining genes; (4) p-value adjusted for multiple comparisons (92 genes): p = 0.131 for FIP1L1, p = 0.735 for STAT5B, and p > 0.999 for remaining genes; (5) p-value adjusted for multiple comparisons (92 genes): p = 0.307 for FIP1L1, p > 0.999 for remaining genes; (6) p-value adjusted for multiple comparisons (92 genes): p = 0.253 for MTAP, p = 0.626 for IFNA1, p = 0.720 for MLLT3*.

In multivariate regression models, CNAs were predicted by following covariates: in *CDKN2A* by high risk (HR) (β = −1.86, *p* = 0.030) with HR decreasing the risk of CNAs; in *FIP1L1, PHF6*, and *WT1* by relapse (respectively, β = 1.67, *p* = 0.046; β = 2.15, *p* = 0.032; and β = 4.66, *p* = 0.036) with relapse increasing the risk of CNAs; in *TRG* by WBC (β = 0.001, *p* = 0.007) with increasing risk of CNAs with growing WBC; in *IFNA1* and *MLLT3* by age (respectively, β = −0.12, *p* = 0.039; and β = −0.18, *p* = 0.011) with decreasing risk of CNAs with growing age (Table 3).

**Table 3:** Variables predictive of CNVs in particular genes in multivariate logistic regression coefficient–β coefficient in logistic regression model with CNVs as the outcome variable.

**Gene**		**Constant**	**Sex, male**	**WBC**	**Response to steroids, good**	**Risk, HR**	**MRD FMC 15 day**	**Relapse**	**Age, years**
*ALK*	Coefficient	2.01	−0.25	0.001	−0.22	−17.4	−0.02	−15.33	−0.02
	*p*	>0.999	0.738	0.213	>0.999	0.997	0.836	0.997	0.862
*CDKN2A*	Coefficient	2.79	0.06	0.001	−0.64	−1.86	−0.01	2.07	−0.07
	*p*	0.033^*^	0.920	0.998	0.462	0.030^*^	0.705	0.068	0.274
*CDKN2B*	Coefficient	1.97	0.05	−0.001	−0.48	−0.58	−0.02	0.71	−0.08
	*p*	0.086	0.930	0.377	0.547	0.459	0.179	0.375	0.166
*CSF1R*	Coefficient	02.49	18.5	−0.001	−18.7	−18.6	0.02	1.01	0.09
	*p*	>0.999	0.995	0.309	0.997	0.997	0.296	0.413	0.435
*FIP1L1*	Coefficient	−1.41	−0.57	0.001	−1.20	0.57	−0.01	1.67	0.04
	*p*	0.328	0.407	0.526	0.217	0.603	0.759	0.046^*^	0.542
*IFNA1*	Coefficient	1.30	0.30	−0.001	−0.58	−0.30	−0.02	0.01	−0.12
	*p*	0.249	0.563	0.487	0.484	0.709	0.219	0.986	0.039^*^
*LEF1*	Coefficient	0.13	−1.41	0.001	−1.34	−1.88	0.06	2.46	−0.001
	*p*	0.994	0.072	0.495	0.994	0.992	0.145	0.178	0.989
*MLLT3*	Coefficient	1.11	0.11	−0.001	−0.23	−0.47	−0.01	0.22	−0.18
	*p*	0.359	0.852	0.346	0.802	0.589	0.566	0.783	0.011^*^
*MTAP*	Coefficient	2.52	−0.06	−0.001	−0.58	−1.52	−0.01	1.69	−0.10
	*p*	0.038^*^	0.907	0.239	0.495	0.067	0.688	0.058	0.073
*NOTCH2*	Coefficient	15.72	0.19	−0.001	−16.81	−17.92	0.03	1.15	−0.02
	*p*	0.993	0.837	0.053	0.993	0.992	0.193	0.344	0.874
*PDGFRB*	Coefficient	−3.25	17.95	−0.001	−18.31	−17.55	0.02	0.23	0.07
	*p*	>0.999	0.996	0.584	0.997	0.997	0.307	0.872	0.577
*PHF6*	Coefficient	−0.68	−0.16	−0.001	−0.85	−1.49	−0.01	2.15	−0.01
	*p*	0.690	0.835	0.364	0.519	0.289	0.644	0.032^*^	0.920
*SLC5A8*	Coefficient	7.11	−0.09	0.001	−9.42	−18.83	0.13	−14.92	−0.16
	*p*	0.999	0.950	0.636	0.998	0.997	0.156	0.997	0.438
*STAT5B*	Coefficient	−0.07	−0.49	−0.004	0.03	−0.01	11.01	−0.42	17.77
	*p*	>0.999	>0.999	0.993	>0.999	0.996	0.993	>0.999	0.994
*TLX3*	Coefficient	13.69	0.36	0.001	−17.01	−15.78	0.01	−0.48	0.02
	*p*	0.994	0.698	0.492	0.993	0.994	0.592	0.701	0.827
*TRG*	Coefficient	1.12	0.08	0.001	0.45	−12.06	0.05	0.30	−0.13
	*p*	0.469	0.911	0.007^**^	0.646	0.255	0.143	0.778	0.115
*WT1*	Coefficient	−24.1	17.4	0.001	0.46	−1.16	0.06	4.66	0.12
	*p*	0.994	0.995	0.256	0.783	0.627	0.115	0.036^*^	0.511

* p < 0.05,

** *p < 0.01. The bold values are considered statistically significant*.

## Discussion

In this study, we performed whole-genome characterization of CNAs in 86 pediatric patients diagnosed with T-ALL. T-cell acute lymphoblastic leukemia can be classified based on both the immunophenotype and genetic alterations. Many of these variations are not mutually exclusive, and together, they lead to dysregulation of several signaling pathways involved in cell maturation, differentiation, proliferation, and apoptosis (8, 9, 11). The deletions/duplications rates in our cohort were similar to those previously described in the literature (9). One of the limitations of this study is lack of information about point mutations and gene fusions in our T-ALL samples, which would place specific CNAs that have been detected in the leukemic genome in the context of the remaining molecular defects.

The SNP array significantly improved the molecular characterization of pediatric ALL (10). Olsson et al. (13) presented 91 and 84% abnormal karyotypes after SNP array analysis of patients with B-cell precursor (BCP)–ALL and T-ALL, respectively. In our study, karyotypes were altered in 77% of pediatric T-ALL cases. Numerical aberrations were present in only 10 patients (single trisomies). In our T-ALL cohort, we found fewer structural abnormalities >10 Mb compared to BCP-ALL, no monosomies, and only segmental LOH. These observations correspond with those of other studies (14). Whole-chromosome Uniparental Disomies (UPIDs) are very rare (<1%) in T-ALL (14, 15), in contrast to pediatric BCP-ALL, where such LOHs are observed in 5% to 10% of cases (16). Moreover, Karrman et al. (15) suggested that deletions in T-ALL are more common [100/128 (78%)] than duplications [28/128 (22%)], which is consistent with our observations. However, repeated structural abnormalities are more common in T-ALL, as is LOH within chromosome 9. Additionally, the most frequent structural changes (>10 Mb) occurred on 9p21.3, as monoallelic or biallelic deletions within 9p21.3.

In two cases, SNP arrays allowed the identification of aberrations that were not detected by conventional karyotyping. The main advantage of SNP arrays is that DNA from tumor cells is used instead of mitotically dividing cells within the cell culture. In our cohort, *NUP214* and *ABL1* regions were amplified in 5 to 6% of T-ALL patients (17). We detected the NUP214-ABL1 extrachromosomal (episomal) amplification of ABL1 and NUP214 and confirmed the findings by FISH. This study showed the impact of the SNP array in the detection of episomal amplification, which is undetectable using routine cytogenetic tests. The amplification of the *NUP214-ABL1* gene on episomes is associated with a higher risk of relapse and chemoresistance in T-ALL. However, aberrantly expressed Abl1 kinase due to *NUP214-ABL1* fusion is a target for therapy with Abl1 inhibitors. Therefore, identifying ABL1 fusions in T-ALL is extremely important from a diagnostic and therapeutic point of view (18).

Moreover, we presented a patient (z219-401) with a chromothriptic-like pattern on chromosome 11. Chromothripsis has been associated with aggressive tumor progression and poor prognosis (19). In childhood, ALL chromothripsis has been described in rare BCP-ALL cases showing intrachromosomal amplification of chromosome 21(iAMP21) (20). The biological and clinical significance of this genome instability involving the other chromosomes in ALL needs further investigation (21).

Regarding abnormalities involving genes acting as transcription factors, *PAX5* was the most frequently altered gene [*n* = 12 (14.0%)], which is in accordance with previous studies (11, 13). *PAX5* plays a key role in the development and maturation of B cells. Inactivation of *PAX5* as a result of deletion or mutation is characteristic of BCP-ALL. However, the loss of *PAX5* function may affect the pathogenesis of T-ALL because B-cell dedifferentiation to the progenitor cell stage with multilinear potential occurs (12, 22). A decrease in *PAX5* expression in T-ALL may also be associated with an increase in promoter methylation of this gene (23).

*RUNX1* genetic alterations are associated with poor prognosis because inactivation of this gene results in inhibition of T-cell transformation (24). The lack or reduced expression of *RUNX1* also affects the activity of *MYB, MYC*, and *GATA3* oncogenes, which confirms the key role of *RUNX1* in the pathogenesis of T-ALL (24). In the described cohort of patients, *RUNX1, MYB*, and *GATA3* changes affected 8.1% (*n* = 7), 12.8% (*n* = 11), and 1.2% (*n* = 1) of cases, respectively, which corresponds to the results that have been previously reported (4, 9, 25).

Deletion of the Wnt signaling central transcription mediator, *LEF1*, was found in 12.8% (*n* = 11) of cases. The activity of this signaling pathway is closely related to the normal course of hematopoiesis; thus, the disturbed expression of key Wnt pathway molecules is characteristic of leukemias (26). Other studies reported a similar frequency of *LEF1* alterations in T-ALL (4, 27, 28). Moreover, Wang and Zhang (29) indicated that lower expression of *LEF1* is associated with ETP-ALL, but we cannot compare our data to published results because of the limited number of ETP-ALL cases (*n* = 2) in our cohort.

The role of the tumor suppressor *PHF6* in the pathogenesis of T-ALL is not fully understood, but it remains one of the most frequently altered genes in this disease. *PHF6* alterations may be associated with glucocorticoid resistance (30). The number of *PHF6* CNAs in the analyzed group was similar to that previously reported (14% vs. 13%) (31). Moreover, Wendorff et al. (32) reported that the promotion of self-renewal capacity of hematopoietic stem cells driven by the loss of *PHF6* stimulates leukemia initiation T-ALL. Thus, *PHF6* deletion may be associated with leukemia development (32). Another negative regulator of the cell cycle is *RB1*; its alterations were detected in 24.4% (*n* = 21) of cases. Another mechanism by which *RB1* activity might also be inhibited in T-ALL is by increased expression of mir-150 (33). We also observed alterations in *MED12* in 2.33% (*n* = 2) of cases. Deletion or mutation within *MED12* oncogene may act as a tumor suppressor by inhibiting chemotherapy-induced apoptosis. This antiapoptotic effect by regulating the TGFβ pathway translates into an increase in resistance to anticancer drugs in the case of repression of *MED12* (34). We also identified CNAs within *JAZF1* in 5.81% of cases (*n* = 5). Chromosomal aberrations involving this gene are associated with the pathogenesis of endometrial stromal tumors, and *JAZF1* itself acts as a transcription repressor (35). To date, no alterations within this gene have been described in the context of hematological diseases, including ALL.

Although *NOTCH1* alterations play a key role in the pathogenesis of T-ALL (36), *NOTCH1* abnormalities were detected in 10.5% (*n* = 9) of the leukemic cases in our cohort. However, this difference may result from the fact that we only analyzed CNAs in the *NOTCH1* and *NOTCH2* genes. In T-ALL cases, mutations within the *NOTCH1* gene occurred much more frequently than NOTCH1 deletions or duplications (36). Impaired *NOTCH1* activity may result in the activation of specific oncogenes (*c-MYC, NFkB*, or mTOR) or inhibition of tumor suppressor expression (*FBXW7, PTEN, CDKN1B*) (37). Therefore, we cannot estimate the impact of alterations within genes of *NOTCH1*-related signaling pathways, as we do not have data on *NOTCH1* mutations (37). Similarly, we observed that *FBXW7* alterations occurred much less frequently in our cohort compared to the frequency found in other studies (3.4% vs. 19–20%) (38, 39). *FBXW7* controls protein turnover by participating in the ubiquitination processes of these molecules and is also a part of the NOTCH signaling pathway. Therefore, the NOTCH signaling pathway is activated most often by genetic alterations within this gene or as a result of loss of function of its negative regulator, *FBXW7*. The results from previous studies do not clearly indicate the prognostic value of *NOTCH1* and *FBXW7* lesions in T-ALL (38–41). Nevertheless, SNP arrays are not appropriate for *NOTCH1* and *FBXW7* mutation status assessment as a single technique, and other sequencing methods need to be used to obtain a complete profile of aberrations in these genes in T-ALL patients.

Activation of Notch1 pathway can also occur as a result of dysregulation within other signaling pathways, such as the PI3K/mTOR pathway. We identified genetic alterations involving the PI3K/mTOR pathway genes in 20 cases (*PTEN*: *n* = 10, *AKT1*: *n* = 6, *PIK3CD*: *n* = 4), which corresponded to the data previously described (42–44). PTEN is a negative regulator of Notch1 pathway, and disturbances in its activity (due to mutation or deletion) may be associated with unfavorable long-term outcomes in some cases of T-ALL (45, 46). Additionally, a change in PTEN expression secondary to NOTCH1-activating mutation may result in cellular resistance to γ-secretase inhibitors (43). A few studies have shown that *PTEN* alterations can affect the risk of relapse; therefore, screening of alterations of this gene could potentially improve risk group stratification (43, 44).

In the study cohort, other signaling pathways, such as the JAK/STAT pathway and RAS pathway, may be affected by CNAs within particular genes. The CNA rates of genes of these pathways are similar to data previously described (31.4% vs. 25% and 11.6% vs. 15%, respectively) (9). Impaired activity of *RAS* occurs as a result of *NRAS* and *KRAS* deletion or *FLT3* duplication. For each of these genes, the alteration rate was similar to that previously described in childhood lymphoblastic leukemia (9).

The interferon α1 gene, *IFNA1*, had a mutation frequency of 39.5% (*n* = 34). Interferons have antiproliferative activity, and their expression influences cancer initiation or progression (47).

We observed an increased frequency of alterations within the genes involved in cell cycle regulation. *CDKN2A/B* deletions [*n* = 62 (72.1%) and *n* = 51 (59.3%), respectively] showed even higher prevalence than that described in other studies. *CDKN2A/B* acts as a tumor suppressor (48, 49). According to our results, only *CDKNA2* deletions were associated with a specific mature immunophenotype of T-ALL (90.5%), in comparison with cortical–T-ALL (73.9%) and pre–T-ALL (68.0%) (*p* = 0.04). Our data also showed a statistically significant co-occurrence of deletions in *CDKN2A* and *LEF, PAX5, TRG*, and *MTAP*, as well as *CDKN2B* and *PAX5, TRG, JAK2*, and *MTAP*. Co-occurrence of *CDKN2A* and *CDKN2B* deletions was reported in 51 cases. Some studies estimated the effect of deletion (mono- or biallelic) of *CDKN2A/B* on outcome, but the results are inconclusive (50). Similar concomitance between particular gene deletions (*WT1, IFNA, RB1*, and *CTCF*) was previously reported by Zhang et al. (51). We did not observe the co-occurrence between *SUZ12* and *CDKN2AB* alterations, although Noronha et al. (4) indicate such concomitance in their study. The study of Karrman et al. (15) revealed that *CDKN2A* deletions were significantly associated with a high WBC count, but we did not find a similar association in our cohort. Interestingly, the co-occurrence of *CDKN2A* deletions with *CDKN2B, MTAP*, or *MLLT3* deletions in the same T-ALL patients was previously reported by Yeh et al. (52). Moreover, our results indicate similar concomitance between *MTAP* and *CDKNAB* deletions. However, in contrast to our results, Yeh et al. (52) reported no significant correlation between the status of CNAs and clinical features, such as sex, age, and WBC.

Deletions and duplications within the genes responsible for posttranscriptional processing play an important role in leukemogenesis. Copy number abnormality rate in this group of genes is comparable to the data described in the literature (9). In our cohort, the most frequently mutated gene in this group was *FIP1L1* [*n* = 18 (20.9%)]. *FIP1L1* status was significantly related to the classical prognostic factors in T-ALL. Alterations in *FIP1L1* were more often associated with a worse response to prednisone and a higher MRD level on day 15 (>10%). To date, no case indicating a relationship between intragenic *FIP1L1* deletion and T-ALL pathogenesis has been described. However, in myeloproliferative disorders with eosinophilia, *FIP1L1-PDGFRBA* fusion has been observed. The FIP1L1-PDGFRA protein is composed of the first 12 exons of *FIP1L1* and from truncated exon 12 (containing the last 17 amino acids) to exon 23 of *PDGFRA*. The FIP1L1-PDGFRA fusion protein is a constitutively activated tyrosine kinase that joins the first 233 amino acids of FIP1L1 to the last 523 amino acids of PDGFRA (53). The presence of such a rearrangement allows the implementation of targeted treatment with the tyrosine kinase inhibitor imatinib (54, 55). In our cohort, the intragenic deletion of *FIP1L1* (exons 5–8 or exons 6–10) was found. To date, there are no published data indicating the role of the *FIP1L1* intragenic deletion in T-ALL pathogenesis.

Inactivation of epigenetic factors results in arresting T-cell development, which was confirmed by studies on ETP-ALL mouse models (56). Furthermore, *WT1* haploinsufficiency may promote the accumulation of somatic mutations or mutations of other epigenetic factors in progenitor cells, leading to tumor transformation (57). Recurrent microduplications in T-ALL are less common. *MYB* (6q23.3) is gained in ~10% of cases as a focal duplication involving solely the *MYB* locus (12). Interestingly, in our study, CNAs in *MYB* exist only in association with *AIH1* alterations, accounting for 12.94% of cases.

## Conclusions

Our analysis shows that the profile of CNAs is associated with the immunophenotypic and clinical features of T-ALL. For the first time, we described the link between *FIP1L1* deletions and the clinical features of patients with T-ALL. Moreover, we observed an increased percentage of cases with concomitant alterations within the *MYB* and *AIH1* genes. We also characterized structural aberrations >10 Mbp, which was lower in our cohort than in BCP-ALL, and we showed that SNP array definitely improved the molecular characterization of pediatric T-ALL. In some specific cases including those harboring *NUP214-ABL1*, SNP array might be valuable diagnostic tool for selecting patients who may benefit from targeted therapy.

## Data Availability Statement

The datasets presented in this study can be found in online repositories. The names of the repository/repositories and accession number(s) can be found below: NCBI GEO under the accession GSE147381.

## Ethics Statement

The studies involving human participants were reviewed and approved by Ethics Committee of Medical University of Silesia. Written informed consent to participate in this study was provided by the participants' legal guardian/next of kin.

## Author Contributions

ML planned the study. ML, AP, and WM were responsible for the conception and design of the study. JZ, ŁS, KS, OH, and TS shared patients' clinical data. BS and JT conducted the laboratory work. ML and MW were responsible for the interpretation of genetic data. MB was responsible for additional statistical analysis. ML, MW, and JZ were responsible for the acquisition of literature for the manuscript. ML and MW wrote the paper. AP, WM, JK, and TS supervised the paper. All authors have read and approved the final manuscript.

## Conflict of Interest

The authors declare that the research was conducted in the absence of any commercial or financial relationships that could be construed as a potential conflict of interest.
