# Supplementary material for: Advantages and Limitations of SNP Array in the Molecular Characterization of Pediatric T-Cell Acute Lymphoblastic Leukemia
Source: Front Oncol. 2020 Jul 17;10:1184. doi: 10.3389/fonc.2020.01184 (PMC7379740; doi:10.3389/fonc.2020.01184)
Supplement: Supplementary file 1 [file Table_1.DOCX]

**Table S1.** The 92 selected genes and their chromosomal positions

| Gene symbol* | Gene name | Position |
| --- | --- | --- |
| *ABL1* | Abelson murine leukemia viral oncogene homolog | 9q34.12 |
| *AGAP2* | ARF GTPase activing protein with GTPase domain | 12q14.1 |
| *AHI1* | Abelson helper integration site 1 | 6q23.3 |
| *AKT1* | v-akt murine thymoma viral oncogene homolog 1 | 14q32.33 |
| *ALK* | Anaplastic lymphoma kinase | 2p23.2-p23.1 |
| *ASXL2* | ASXL transcriptional regulator 2 | 2p23.3 |
| *BAZ1A* | Bromodomain adjacent to zinc finger domain 1A | 14q13.1-q13.2 |
| *BCL11B* | B-cell CLL/lymphoma 11B (zinc finger protein) | 14q32.2 |
| *CCND2* | cyclin D2 | 12p13.32 |
| *CCND3* | cyklin D3 | 6p21.1 |
| *CDKN1B* | cyklin-dependent kinase inhibitor 1B | 12p13.1 |
| *CDKN2A* | cyclin-dependent kinase inhibitor 2A | 9p21.3 |
| *CDKN2B* | cyclin-dependent kinase inhibitor 2B | 9p21.3 |
| *CNOT3* | negative regulator of transcription 3 | 19q13.42 |
| *CREBBP* | CREB binding protein | 16p13.3 |
| *CSF1R* | colony- stimulating factor1; receptor | 5q32 |
| *CTCF* | CCCTC-binding factor (zinc finger protein) | 16q22.1 |
| *DDX3X* | Dead / H Box3, X-linked | Xp11.4 |
| *DHX15* | Deah box polipeptide 15 | 4q15.2 |
| *DNM2* | dynamin 2 | 19p13.2 |
| *EPOR* | Erythropoietin receptor | 19p13.2 |
| *ETV6* | ets variant 6 | 12p13.2 |
| *EZH2* | enhancer of zeste homolog 2 (Drosophila) | 7q36.1 |
| *FAT1* | FAT atypical cadherin 1 | 4q35.1 |
| *FBXW7* | F-box and WD repeat domain containing 7, E3 ubiquitin protein ligase | 4q31.3 |
| *FLIP1L1* | FIP1-like | 4q12 |
| *FLT3* | fms-related tyrosine kinase 3 | 13q12.2 |
| *GATA3* | GATA binding protein 3 | 10p14 |
| *HOXA11* | homeobox A11 | 7p15.2 |
| *HUWE1* | HECT, UBA and WWe domains-containig protein | Xp11.22 |
| *IFNA1* | Interferon alpha-1 | 9p21.3 |
| *IL7R* | interleukin 7 receptor | 5p13.2 |
| *IKZF1* | IKAROS family zinc finger 1 (Ikaros) | 7p12.2 |
| *JAK1* | Janus kinase 1 | 1p31.3 |
| *JAK2* | Janus kinase 2 | 9p24.1 |
| *JAK3* | Janus kinase 3 | 19p13.11 |
| *JAZF1* | Juxtaposed with another zinc finger gene1 | 7p15.1-p15.1 |
| *KDM6A* | Lysine-specific demethylase 6A | Xp11.3 |
| *KMT2A* | Lysine-specific methyltransferase 2A | 11q23.3 |
| *KMT2C* | Lysine-specific methyltransferase 2C | 7q36.1 |
| *KMT2D* | Lysine-specific methyltransferase 2D | 12q13.12 |
| *KRAS* | v-Ki-ras2 Kirsten rat sarcoma viral oncogene homolog | 12p12.1 |
| *LEF1* | lymphoid enhancer-binding factor 1 | 4q25 |
| *LMO2* | LIM domain only 2 (rhombotin-like 1) | 11p13 |
| *LYL1* | lymphoblastic leukemia derived sequence 1 | 19p13.2 |
| *MED12* | Mediator complex subunit 12 | Xq13.1 |
| *MLLT3(AF9)* | MLLT3 super elontagion complex subunit | 9p21.3 |
| *MTAP* | Methylthioadenosine phosphorylase | 9p21.3 |
| *MYB* | v-myb myeloblastosis viral oncogene homolog (avian) | 6q23.3 |
| *MYCN* | V-myc avian myelocytomatosis viral-related oncogene | 2p24.3 |
| *NOTCH1* | notch 1 | 9q34.3 |
| *NOTCH2* | Notch 2 N-terminal-like R | 1p12 |
| *NRAS* | neuroblastoma RAS viral (v-ras) oncogene homolog | 1p13.2 |
| *NTRK3* | Neurotrophic tyrosine kinase receptor | 15q25.3 |
| *NUP214* | Nucleoporin, 214-KD | 9q34.13 |
| *ORAI1* | ORAI calcium release-activated calcium modulator 1 | 12q24.31 |
| *PAK2* | P21 protein-activated kinase 2 | 3q29 |
| *PAX5* | paired box 5 | 9p13.2 |
| *PBX1* | preB-cell leukemia transcription factor 1 | 1q23.3 |
| *PDGFRB* | Plateled-drived growth factor receptor beta | 5q32 |
| *PHF6* | PHD finger protein 6 | Xq26.2 |
| *PIK3CA* | phosphatidylinositol-4,5-bisphosphate 3-kinase, catalytic subunit alpha | 3q26.32 |
| *PIK3CD* | Phosphatidylinositol 3-kinase, catalytic delta | 1p36.22 |
| *PIK3R1* | Phosphatidylinositol 3-kinase, regulatory subunit 1 | 5q13.1 |
| *PML* | Acute promyelocytic lekemia, inducer OF | 15q24.1 |
| *PTEN* | phosphatase and tensin homolog | 10q23.31 |
| *PTPN2* | protein tyrosine phosphatase, non-receptor type 2 | 18p11.21 |
| *PTPN11* | protein tyrosine phosphatase, non-receptor type 11 | 12q24.13 |
| *RAG1* | Recombination-activating gene 1 | 11p12 |
| *RAG2* | Recombination-activating gene 1 | 11p12 |
| *RB1* | retinoblastoma 1 | 13q14.2 |
| *ROBO1* | Round about guidance receptor 1 | 3p12.3 |
| *RPL10* | Ribosomal protein L10 | Xq28 |
| *RPL5* | Ribosomal protein L5 | 1q22.1 |
| *RBPJ* | recombination signal binding protein for immunoglobulin kappa J region | 4p15.2 |
| *RELN* | reelin | 7q22.1 |
| *RUNX1* | runt-related transcription factor 1 | 21q22.12 |
| *SET* | SET nuclear protooncogene | 9q34.11 |
| *SETD2* | SET domain containing 2 | 3p21.31 |
| *SLC5A8* | Solute carrier family 5, (iodide transporter) member 8 | 12q23.1-q23.2 |
| *SMARCA4* | SWI/SNF related, matrix associated, actin dependent regulator of chromatin, subfamily A, member 4 | 19p13.2 |
| *SMARCC1* | SWI/SNF related, matrix associated, actin dependent regulator of chromatin, subfamily C, member 1 | 3p21.31 |
| *SORBS2* | Sorbin and SH3 domains-containing protein 2 | 4q35.1 |
| *ST13P4* | Suppression of Tumorigenicity 13 Pseudogene 4 | 13q14.2 |
| *STAT5B* | Signal transducer and activator of transcription 5B | 17q21.2 |
| *STIL* | SCL/TAL1-interrupting locus | 1p33 |
| *SUZ12* | suppressor of zeste 12 homolog (Drosophila) | 17q11.2 |
| *TAL1* | T-cell acute lymphocytic leukemia 1 | 1p33 |
| *TCF3* | transcription factor 3 | 19p13.3 |
| *TRG* | T-cell receptor gamma chain constant region 1 | 7p14.1 |
| *TSPYL2* | TSPY-like 2 | Xp11.22 |
| *U2AF1* | U2 small nuclear RNA auxiliary factor 1 | 21q22.3 |
| *USP7* | Ubiquitin-specific protease 7 | 16p13.2 |
| *USP9X* | Ubiquitin-specific protease 9, X-linked | Xp11.4 |
| *WT1* | Wilms tumor 1 | 11p13 |

*HGNC approved gene symbol
