# Supplementary material for: Advantages and Limitations of SNP Array in the Molecular Characterization of Pediatric T-Cell Acute Lymphoblastic Leukemia
Source: Front Oncol. 2020 Jul 17;10:1184. doi: 10.3389/fonc.2020.01184 (PMC7379740; doi:10.3389/fonc.2020.01184)
Supplement: Supplementary file 2 [file Table_2.DOCX]

| **UPN**  **of Patient** | **Sex** | **Age** | **Follow up** | **Cytogenetic karyotype** | ***KMT2A*** | ***BCR/ABL1*** | **Molecular Karyotype > 10Mb** |
| --- | --- | --- | --- | --- | --- | --- | --- |
| 1478 | F | 3.6 | alive | 46,XX,del(6q23) | N | N | arr[GRCh37]6q14.1q21(79110264_110247755)x1, 9p24.3p13.1(191128_40087758)hmz |
| Y02335 | F | 7.7 | alive | 46,XX | N | N | arr[GRCh37](13)2-3,17p13.3p11.2(1070585_18070728)x1-2, 17q11.2q25.3(31329329_81041938)x2-3 |
| 907 | F | 5.6 | alive | 46,XX,t(7;10)(q34;q24),inc | N | N | arr[GRCh37](8)x2~3,9p23q13(9009351_67983174)x1,19p13.11q12(17795314_29189724)x2-3 |
| E00621 | M | 14.2 | alive | 46,XY | N | N | arr(1-22)x2,(X,Y)x1 |
| Y02167 | M | 9.8 | **relapse/death** | 46,XY,t(1;8)(q32;q24)/46,  sl,t(9;9)(q10;q10) | N | N | arr(1-22)x2,(X,Y)x1 |
| 1100* | M | 11.5 | alive | 46,XY | N | N | arr[GRCh37]1p36.33p36.31(849466_6445105)x1-2,1p36.22p31.1(10387929_76236452)x2-3,1p31.1p12(79915304_120001330)x1-2,1q21.2q44(148514235_249224684)x2-3,5q31.1q35.3(130697570_180719789)x1-2,7q31.1q36.3(108354488_159119707)x1-2,9p23p21.1(10407804_32282978)x1,9p21.1p13.2(32158191_38326473)x2-3,9q21.11q33.2(71512667_123009219)x1-2,9q33.2q34.3(124629919_141020389)x2-3,13q13.3q21.33(40010662_70747804)x1 |
| 5190 | F | 4.8 | alive | 46,XX | N | N | arr(1-22,X)x2 |
| Y01486 | M | 10.3 | alive | 46,XY | N | N | arr[GRCh37]6q12q15(64385581_81861581)x1,9p24.3p21.3(192128_21818011)hmz |
| E01830 | M | 8.5 | alive | 46,XY | N | N | arr[GRCh37]9p24.3p21.2(192128_25954668)hmz |
| 1074 | M | 3.4 | alive | 46,XY | N | N | arr[GRCh37]9p24.3p13.3(192128_35518623)hmz |
| 684 | F | 17.5 | alive | 46,XX | N | N | arr[GRCh37]8p23.3p12(158048_35574307)x1-2,8p11.21q24.3(40829442_146295771)x2-3, 9q21.11q34.3(71742712_141020389)x3, 9p24.3p13.2(192128_37964848)hmz,(21)x2-3, (22)x2-3, (X)x4 |
| 6982 | F | 5.0 | alive | 46,XX | N | N | arr(1-22,X)x2 |
| 1056 | M | 15.1 | alive | 46,XY | N | N | arr(1-22)x2,(X,Y)x1 |
| YSS | M | 4.5 | **relapse/death** | no result | N | N | arr[GRCh37]6q12q21(69471042_110789107)x1-2,13q14.1q31.2(48749292_89895472)x1 |
| YED | M | 4.5 | alive | 46,XY | N | N | arr(1-22)x2,(X,Y)x1 |
| z369_401 | M | 2.7 | **relapse** | 46,XY,t(3;14;10)(q21;q11.2;q22)del(9)(p13) | N | N | arr[GRCh37]9p24.3q13(203861_67983174)x1 |
| Y5D | M | 7.9 | alive | 46,XY | N | N | arr[GRCh37]9p24.3p13.1(191128_40087758)hmz |
| 1545 | M | 5.2 | alive | 46,XY | N | N | arr[GRCh37]7q34q36.3(140528073_159119707)x1-2,9p24.321.1(192128_33190239)hmz,11p15.5p13(230615_33601913)x2-3 |
| Y01849 | F | 17.9 | alive | 46,XX | N | N | arr[GRCh37]12p13.31p11.23(9518980_27089633)x1-2 |
| 993 | F | 3.2 | alive | 46,XX,del(6)(q13q22) | N | N | arr[GRCh37]6q12q21(66135197_107652825)x1-2 |
| Y00_719 | F | 10.7 | alive | 46,XY | N | N | arr[GRCh37]11p15.5p12(198509_40710263)hmz |
| 6745 | F | 14.9 | alive | 46,XX | P | N | arr(1-22,X)x2 |
| Y01972 | F | 8.0 | **death** | 46,XX | N | N | arr(1-22,X)x2 |
| E01130 | M | 3.9 | alive | 46,XY | N | N | arr(1-22)x2,(X,Y)x1 |
| YQW | M | 6.3 | alive | 46,XY | N | N | arr[GRCh37]7q33q36.3(134012746_159119707)x1, 8q24.11q24.3(119137488_146295771)x3,12p13.33p11.22(173786_30186386)x1 |
| 6079 | M | 10.5 | alive | 46,XY,t(9;11)(p22;q23) | P | N | arr(1-22)x2,(X,Y)x1 |
| Y01701 | F | 2.4 | alive | 46,XX | N | N | arr[GRCh37]9p24.3p21.1(203861_28186953)x1 |
| 6768* | M | 15.3 | alive | 46,XY,del(5)(q31q35)del7(q32q36)del12(p12p13)+mar | N | N | arr[GRCh37] 1p36.33p35.2(849466_32072406)x1-2, 5q14.3q35.3(83250917_180719789)x1-2, 7p22.3p15.2(43360_27358452)x2-3,7q21.3q36.3(93087151_159119707)x1-2, 12p13.33p11.21(173786_31598670)x1-2 |
| 1553** | M | 8.4 | alive | 46,XY | N | N | arr(1-22)x2,(X,Y)x1 |
| 541 | M | 10.7 | **relapse/death** | 46,XY,t(7;14;19) | N | N | arr(1-22)x2,(X,Y)x1 |
| 159** | M | 4.7 | alive | 92,XYY/46,XY | N | N | arr(1-22)x2,(X,Y)x1 |
| 6066 | M | 4.8 | alive | 46,XY | N | N | arr(1-22)x2,(X,Y)x1 |
| EOO206 | M | 14.8 | alive | 46,XY | N | N | arr[GRCh37] 5q13.3q15(73945737_95945334) hmz |
| YOO_615* | M | 11.6 | **relapse** | 45,t(X;13)(q26;q14),Y,del(1)(p32),del(5)(q31),-9,del(9)(p16),del(12)(p13),der(18)t(X;9;18),(q26;q32;q21),der(21)t(19;21)(p13;p11) | N | N | arr[GRCh37] 5q31.1q35.3(132822822_180719789)x1-2,9p23q31.3(9022549_114045442)x1,9q33.1q34.3(118514469_141020389)x2-3, 12p13.2p12.1(11256830_22116241)x1-2, 13q13.3q14.3(40004687_54389002)x1-2, 15q13.1q25.2(30290305_82440990)x1-2, 16q21q24.3(57448597_90155062)x1-2, 18q22.1q22.3(61745508_72519390)x1-2, 19p13.3q11(260911_28299209)x2-3 |
| Y00_475 | M | 9.6 | alive | 46,XY,t(1;5)(q32;q35)t(4;14)(q21;q13) | N | N | arr(1-22)x2,(X,Y)x1 |
| 865 | F | 15.2 | alive | 46,XX | N | N | arr(1-22,X)x2 |
| 1025 | M | 7.3 | alive | 46,XY | N | N | arr[GRCh37]8q21q24.3(131101950_146295771)2-3 |
| YOO303 | M | 14.1 | alive | no result | N | N | arr[GRCh37] 16p13.3p11.2(85880_ 32538293)x3,16p11.2q24.3(34197491_90155062)x1 |
| Y7W | M | 7.8 | alive | 45,XY,dic(15;16)(p13;q22)/46,XY | N | N | arr[GRCh37] 16p11.1q24.3(34879032_90155062)x1-2 |
| YOO_724 | M | 12.2 | **death** | 46,XY | N | N | arr(1-22)x2,(X,Y)x1 |
| z225_401 | M | 13.6 | alive | 46,XY,t(11;19)(q23;p13.3) | P | N | arr[GRCh37]9p24.3p13.3(192128_33794124)hmz |
| 929 | M | 4.9 | **relapse/death** | 46,XY | N | N | arr[GRCh37](1p)x1~2(1q)x2~3,(2)x1~2,(3,4,5)x2~3,(6)x3,(7,8)x2~3,(9)x1~2,(10,11,12)x2~3,(13)x1~2,(14)x3,(15)x1~2,(16,17,18)x2~3,(19)x1~2,(20)x2~3,(21)x3,(22)x1~2,(X)x1~2,(Y)x0~1 |
| Y02359 | F | 8.0 | alive | 46,XX,del(6)(q13q22) | N | N | arr[GRCh37] 6q12q22.1(69246088_115523584)x1-2,9p24.3p13.3(192128_36152153) hmz |
| 1117 | M | 14.1 | alive | 46,XY | N | N | arr(1-22)x2,(X,Y)x1 |
| 1725 | M | 4.0 | alive | 46,XY | N | N | arr(1-22)x2,(X,Y)x1 |
| 8514 | F | 6.7 | alive | 46,XX | N | N | arr[GRCh37]15q24.3q26.3(76895760_102345504)x3 |
| 1475 | M | 7.6 | alive | 46,XY | N | N | arr[GRCh37] 9p24.3p21.2(203861_26085449)x1, 9p13.3q13(34888026_67983174)x1 |
| 659 | F | 3.3 | alive | no result | N | N | arr[GRCh37] 9p24.3p13.1(191128_40087758)hmz |
| 1476 | F | 16.3 | alive | 46,XX | N | N | arr[GRCh37] 9p24.3p21.1(192128_28321635)hmz,(14)x2-3, 14q23.2q32.33(64475196_106826231) hmz |
| 194 | F | 14.8 | alive | 92,XXXX/del(6q13q23.3) | N | N | arr[GRCh37] 9p24.3p21.3(192128_21854199)hmz,14q11.2q13.2(22641679_36494811)x1-2, 20p11.22q11.23(21575231_35946529)x2-3 |
| YWG** | M | 5.8 | alive | 46,XY | P | N | arr(1-22)x2,(X,Y)x1 |
| 1138 | M | 14.2 | alive | 46,XY | N | N | arr[GRCh37] 18q11.1q23(18540833_78014582) hmz |
| YPK | M | 5.4 | **death** | 46,XY | N | N | arr[GRCh37]5q35.1q35.3(170765559_180719789)x1,9p24.3p22.1(192128_19705636)hmz, |
| 1564 | M | 15.7 | no | 46,XY | N | N | arr[GRCh37] (19)x2-3 |
| YOO439 | F | 5.1 | no | 46,XX | N | N | arr[GRCh37] 5q35.1q35.3(170731940_180719789)x1, 7q36.1q36.3(148013257_159119707)x3, (8)x3 |
| 5412 | M | 3.1 | no | 46,XY | N | N | arr[GRCh37] 6p11.1q16.3(58759014_101059629)x1, 9p24.3p13.2(192128_36815056) hmz |
| 682 | F | 7.1 | no | 46,XX | P | N | arr(1-22,X)x2 |
| z370_401 | F | 3.1 | **death** | 46,XX | N | N | arr[GRCh37] 6q11.1q21(62559639_111657139)x1-2, 9p24.3p21.3(192128_21933125) hmz |
| Y01358 | F | 15.8 | alive | 46,XX | N | N | arr(1-22,X)x2 |
| Y87 | M | 9.2 | alive | 46,XY | N | N | arr[GRCh37] 9p22.2p21.1(17328425_28155249)x1-2 |
| 1301 | M | 2.8 | alive | no result | N | N | arr[GRCh37] 9p24.3p13.2(192128_37964848) hmz |
| Y01980 | M | 13.6 | alive | 46,XY,t(6;11)(q27;q23)/92,XXYY,t(6;11) | P | N | arr(1-22)x2,(X,Y)x1 |
| 1067** | M | 6.4 | alive | 47,XY,+8 | N | N | arr[GRCh37](8)x2~3 |
| 986* | M | 6.4 | alive | 46,XY | N | N | arr[GRCh37]1p36.33p36.22(854277_10485206)x1-2,1p31.1p12(78474239_119195648)x1-2, 5q15q22.1(98095858_110779981)x1-2,5q23.1q35.3(121335741_180719789)x1-2, 13q14.11q33.3(42666962_109761489)x1-2, |
| E01468 | M | 17.7 | **death** | 46,XY | N | N | arr[GRCh37]9p24.3p21.1(191128_32435701)hmz |
| Y01966 | F | 9.8 | alive | 46,XX | N | N | arr[GRCh37]5p15.33p12(113576_44151712)x2-3,9p24.3p13.1(203861_38776786)x1, 18q12.2q21.32(35821707_58993087)x1-2 |
| Y01604 | M | 8.5 | alive | 46,XY | N | N | arr[GRCh37]2p24.2p23.2(18391549_29438888)x1-2, 19p13.3(2733942_5062547)x1, 19p13.2(10814875_12745526)x1 |
| 1200 | M | 2.8 | alive | 46,XY,t(11;19)(q23;p13.3) | P | N | arr(1-22)x2,(X,Y)x1 |
| YAR | M | 4.8 | alive | no result | N | N | arr[GRCh37]5p15.33p13.2(113576_37869941)x2-3,9p24.3p13.2(676264_36708464)x1, 14q11.2q13.3(20511672_36950791)x1-2 |
| 856 | F | 10.9 | alive | 46,XX,t(11;14)(p11.3-15;q11) | N | N | arr[GRCh37] 9p24.3p21.1(192128_32984072) hmz |
| Y02140* | M |  | alive | 46,XY | N | N | arr[GRCh37]3p26.3p24.3(61891_17834822)x1-2,4p16.3p16.1(68345_10453310)x2-3, 4q23q34.1(100986663_173422510)x1,5q15q35.3(96226323_180719789)x1-2, 6q15q27(90668614_170919482)x2-3,(7)x2-3,9p24.3q21.11(203861_71039044)x1-2, 17p13.3p11.2(525_19721156)x1-2,17q11.2q25.3(27290305_81041938)x2-3, 21q22.11q22.3(35786644_48097372)x2-3 |
| 1309 | M | 4.4 | **death** | 46,XY | N | N | arr(1-22)x2,(X,Y)x1 |
| 7657* | M | 6.1 | **death** | 45,XY,-1,dup(1)?(p32p34),der(3)t(1;3)(q?12;q?26),del(5)(q?33), der(11)t(1;11)(p?32;p15),del(12)(p13),der(16)t(?;16)(?;p?13)[44]/46,XY[38] | N | N | arr[GRCh37]1p36.33p36.23(849466_8096240)x1,1p36.23p31.3(8931529_67365806)x3,1p31.12q21.1 (70493564_145289186)x1,3q22.1q29 (133476890_197851986)x1,4q31.3q35.2(155500158_190957473)x3, 5q21.1q35.3(100821228_180719789)x1,6q22.1q27(115144178_170919482)x3, 9p24.3p21.1(203861_28849504)x1,10p15.3p11.1(100026_38258848)x3, 11p15.5p13(230615_35363338)x1,12p13.33p12.1(173786_22885159)x1, 16p13.3p13.2(85880_10023421)x1 |
| 5741 | M | 3.8 | alive | 47,XY,i(9q)(q10;q10),+21[8]/46,XY | N | N | arr[GRCh37] 9p24.3p13.1(203861_38772005)x1, 9q21.12q34.3(72741589_141020389)x2-3,(21)x3 |
| 975 | M | 12.8 | alive | 46,XY | N | N | arr[GRCh37] 6q13q21(74525814_114351627)x1-2, 16q21q24.3(57712418_88818573)x1-2 |
| 163 | M | 13.0 | alive | 46,XY | N | N | arr[GRCh37] 9p24.3p13.3(192128_35714218) hmz |
| Y02265** | M | 8.4 | alive | 46,XY | N | N | arr[GRCh37] 9p23p13.1(10585429_38759982)x1-2 |
| 1606 | M | 1.1 | alive | 46,XY | N | N | arr[GRCh37] 10p15.3p11.21(95661_37275406) hmz |
| 1733 | M | 5.2 | alive | 46,XY, t(11;14)?(p15;q11)[8]/46,XY | N | N | arr(1-22)x2,(X,Y)x1 |
| z219_401 | F | 1.5 | alive | 46,XX,t(6;7)(q23;q22),trp(11)(q13q12) | N | N | arr[GRCh37] 11q14.1q14.3(78827894_88681711)x1,11q14.3q22.1(88681765_98309289)x4, 11q22.1q22.3(99382886_104235560)x4, 11q22.3(104666375_106478262)x3, 11q22.3q23.2(110352530_114453501)x4, 11q23.3(114660848_120894144)x4, 11q24.1q24.2(121810230_126482328)x3, 11q24.2q25(126812620_131759393)x4, 11q25(132355335_132568430)x3, 11q25(132862135_134938470)x3 |
| 920 | M | 10.2 | **relapse/death** | 46,XY | N | N | arr[GRCh37] 10p15.3p11.21(95661_37275406) hmz |
| Y01588 | M | 10.5 | alive | 46,XY | N | N | arr[GRCh37] 1p21.3q21.1(98928270_143932350)x1-2, 9p24.3p21.3(192128_21894495) hmz, 9p21.3p13.3(24005997_35887030) hmz, 12p13.33p12.3(173786_18815997)x1-2 |
| z793_401 | F | 3.5 | **relapse/death** | 46,XX,del(6)(q13q23) | N | N | arr[GRCh37] 4p16.3p15.1(68345_34350170)x1-2, 6q13q22.1(70901584_117241384)x1-2, 7q21.11q36.3(85615814_159119707)x2-3, |
| YOO349 | M | 5.9 | **relapse** | no result | N | N | arr[GRCh37] 19p13.3p11(260911_24505637) hmz |
| z342_401 | F | 1.5 | **relapse/death** | 47,XX,+mar[3]/46,XX[14] | N | N | arr[GRCh37] 9p24.3p21.3(192128_21828110) hmz, 9p21.3p13.2(22041997_38187709) hmz |
| 6482 | M | 5.8 | alive | 46,XY | N | N | arr(1-22)x2,(X,Y)x1 |

**Table 2.** Original karyotypes and revisions based on SNP array analysis in 86 pediatric patients with T-ALL.

The selection criteria included the quality of the frozen diagnostic material, having at least 40% blast cells, and patients aged ≤18 years at the time of T-cell ALL diagnosis. The exclusion criteria were samples collected in heparin and DNA of insufficient quality. A single asterisk (*) corresponds to cases with complex karyotypes (>3 chromosomal aberrations). Double asterisk (**) corresponds to cases with poor quality diagnostic material, with less than 40% blast cells. Abbreviations: F, female; M, male; P, positive by FISH method; N, negative by FISH method.
